# Combination Therapy With Lenvatinib and Radiofrequency Ablation for Patients With Intermediate-Stage Hepatocellular Carcinoma Beyond Up-To-Seven Criteria and Child–Pugh Class A Liver function: A Pilot Study

**DOI:** 10.3389/fonc.2022.843680

**Published:** 2022-05-04

**Authors:** Feiqian Wang, Kazushi Numata, Satoshi Komiyama, Haruo Miwa, Kazuya Sugimori, Katsuaki Ogushi, Satoshi Moriya, Akito Nozaki, Makoto Chuma, Litao Ruan, Shin Maeda

**Affiliations:** ^1^ Ultrasound Department, The First Affiliated Hospital of Xi’an Jiaotong University, Xi’an, China; ^2^ Gastroenterological Center of Yokohama City University Medical Center, Yokohama, Japan; ^3^ Chemotherapy Department of Yokohama City University Medical Center, Yokohama, Japan; ^4^ Division of Gastroenterology of Yokohama City University Graduate School of Medicine, Yokohama, Japan

**Keywords:** radiofrequency ablation, lenvatinib, hepatocellular carcinoma, treatments, intermediate-stage, up-to-seven criteria

## Abstract

**Background:**

The present study aimed to evaluate the efficacy and safety of combined lenvatinib (first-line systemic therapy) and radiofrequency ablation (RFA) therapy in patients with intermediate-stage hepatocellular carcinoma with beyond up-to-seven criteria and Child–Pugh Class A liver function (CP A B2-HCC).

**Methods:**

Twenty-two patients with CP A B2-HCC were enrolled in the study. The patients had no history of systemic treatment. For the initial lenvatinib administration in this study, all of the patients had an adequate course of treatment (no less than two weeks) and were administered the recommended dose. Of them, 13 were treated by means of lenvatinib monotherapy (monotherapy group), while the 9 patients with no contraindication to RFA operation and who had consented to RFA received initial lenvatinib plus subsequent RFA (combination group). The clinical outcomes that were considered to evaluate the treatments included tumor response, prognosis (recurrence and survivals), and possible adverse events (serum liver enzymes and clinically visible complications).

**Results:**

The combination group exhibited a higher object response rate (9/9, 100%) as best tumor response than the monotherapy group (10/13, 76.9%). Longer progression-free survival (PFS) (12.5 months) and overall survival (OS) (21.3) were demonstrated in the combination group than in the monotherapy group (PFS: 5.5 months; OS:17.1 months). The combination group achieved a higher PFS rate (1-year: 74.1%) and OS rate (2-year: 80%) than the monotherapy group (1-year PFS rate: 0%; 2-year OS rate: 25.6%; for PFS, p<0.001; for OS, p=0.022). The treatment strategy was the independent factor for PFS (HR: 18.215 for monotherapy, p =0.010), which was determined by Cox regression analysis, suggesting that a combination strategy may reduce tumor progression when compared to the use of lenvatinib alone. There were no statistically significant intergroup differences that were observed in terms of adverse events, with the exception of ALT elevation (p=0.007) in the combination group.

**Conclusion:**

Our newly proposed combination therapy may potentially be effective and safe for CP A B2-HCC beyond up-to-seven criteria. A larger scale, multicenter, prospective study is warranted to confirm our findings.

## 1 Introduction

Transarterial chemoembolization (TACE) is the standard treatment for Barcelona Clinic Liver Cancer (BCLC) intermediate-stage hepatocellular carcinoma (HCC) (1). However, in real-world clinical practice, the TACE procedure is not as easy to perform as we would expect. For some patients, the pattern of the blood vessels makes it difficult to place the catheter when carrying out TACE treatment. In this setting, no universal consensus for TACE treatment exists (2). More importantly, there is the severe problem of the refractoriness or unsuitability of TACE for patients with large and multifocal tumors (corresponding to lesions beyond up-to-seven criteria), when repeat TACE would be required. Beyond up-to-seven criteria means largest tumor diameter [in cm] + tumor number > 7. In theory, TACE increases tumor hypoxia and activates hypoxic response signaling, thereby inducing the upregulation of the vascular endothelial growth factor (VEGF) and the fibroblast growth factor (FGF), which can lead to tumor revascularization and progression (3). In addition, some HCCs do not show lipiodol uptake, which may result in the treatment being less effective (4). Therefore, complete response (CR) to TACE (especially for repeat TACE treatments) and good survival outcomes are hard to achieve for HCC cases at the higher substages of the intermediate stage (5). From intermediate-stage Substage 1 (B1) to intermediate-stage Substage 2 (B2), and even to intermediate-stage Substage 3 (B3), the reported median survival decreases remarkably (44.8 months, 21.5 months. and 11.3 months, respectively, p<0.001) (6). What is worse, after several TACE treatment sessions, some patients exhibit a deterioration in liver function (7). Statistically, liver function after both the third and fifth TACE sessions differed significantly from that after the first TACE session (first–third, p = 0.0020; first–fifth, p = 0.0008; third–fifth, p = 0.6145) (7).

Systemic therapy has been tried as a first-line treatment for intermediate-stage HCC, especially for larger (≥5 cm) or multiple lesions (mainly indicated for beyond up-to-seven HCC) (8, 9). Very recently, BCLC group have recommended systemic treatment rather than TACE treatment for the patient population with intermediate-stage BCLC with a high tumor burden [“diffuse, infiltrative, extensive HCC liver involvement” (10) and “large multifocal HCC involving both lobes” (11)]. Regarding systemic therapy, lenvatinib is a newly developed oral multi-tyrosine kinase inhibitor (TKI) that results in the dual inhibition of both the VEGF and FGF pathways (12). Based on the results of the REFLECT trial, lenvatinib has been widely approved as a first-line treatment for unresectable HCC in Japan, the United States, Europe, and other parts of Asia (13). Lenvatinib has exhibited favorable results in terms of inhibiting tumor angiogenesis and tumor growth (or in decreasing the size of the lesion) (14) and is more cost-effective than the current mainstay systemic therapy, sorafenib (15). Excitingly, in terms of intermediate-stage HCC, recent research with large multicenter randomized trials has consistently revealed the attractive survival advantage of lenvatinib over sorafenib and TACE treatment. The data showed that the median overall survival (OS) time for lenvatinib treatment was 13.6 months, while for sorafenib, it was 12.3 months (12); the median progression-free survival (PFS) time was 5.8, 3.2, and 2.4 months in the lenvatinib, sorafenib, and TACE groups, respectively (p <0.001) (16). The 6-, 12-, 18-, and 24-month cumulative survival rates in patients treated with lenvatinib were much higher than those in patients who received TACE treatment [p < 0.001) (17)]. Likewise, radiofrequency ablation (RFA), a standard therapy for early-stage HCC, was successfully tried for intermediate-stage HCC by some researchers (18, 19). Its beneficial survival effects were attributed to the complete destruction of the entire tumor. However, there were also problems. First, the thermal effect was considered to be weakened by the heat-sink phenomenon of inter- and peri-tumoral hypervascularity (19). Second, as a recent large-sample multicenter study pointed out, the survival benefit of RFA is limited to B1-and B2-HCC patients, not B3- and B4-HCC patients with poor liver function (20).

Inspired by recently published research on successful experiences and potential problems with the application of lenvatinib and RFA alone for the treatment of intermediate-stage HCC, we attempted to explore better therapy for Child–Pugh Class A (CP A) B2-HCC (characterized by a heavy tumor burden and relatively good liver function) other than TACE. In detail, we planned a retrospective study by introducing a combination of (first-line systemic treatment) lenvatinib and (sequential) RFA for CP A B2-HCC, with the belief that lenvatinib may decrease the vascularity and/or the size of the lesions and that would then RFA destroy the lesions, possibly to a curative degree. To evaluate the efficacy of this combination therapy more objectively, we used lenvatinib monotherapy as a control group. To date, the present study is the first to explore the efficacy and safety of lenvatinib plus RFA treatment for intermediate-stage HCC.

## 2 Materials and Methods

### 2.1 Patient Enrollment

**Figure 1:**
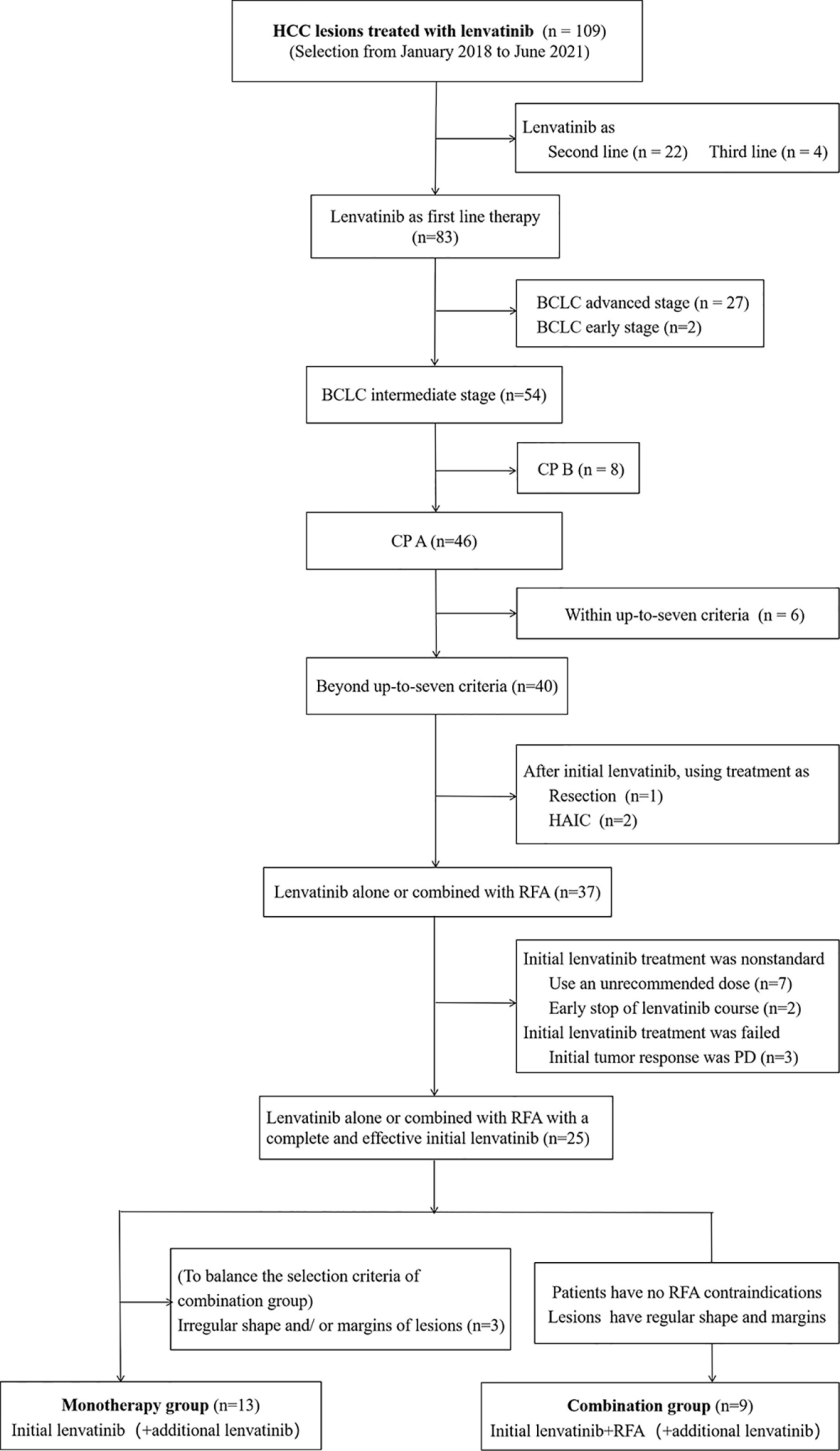
Flowchart of the study population. HCC, hepatocellular carcinoma; BCLC, Barcelona Clinic Liver Cancer; CP, Child–Pugh; HAIC, hepatic arterial infusion chemotherapy; RFA, radiofrequency ablation; PD, progressive disease.

From January 2018 to June 2021, we retrospectively enrolled 109 consecutive adult patients with HCC lesions that were unresectable and scheduled to be treated using lenvatinib. All of the patients were local Japanese people and had been diagnosed with HCC at Yokohama City University Medical Center (YCUMC). HCCs were confirmed by biopsy, cytology, dynamic computed tomography (CT), or magnetic resonance imaging (MRI) examination based on Japanese Society of Hepatology Guidelines (21). It was explained to all of the patients who were enrolled in the study that it would be difficult to treat all of the lesions or larger lesions with the standard therapy, TACE. It was also explained that lenvatinib treatment, with or without RFA, might be better than TACE for achieving tumor response and survival, but this effect had not been completely proven yet. Meanwhile, the patients were also fully informed of the efficacy, potential adverse events (AEs), and costs of lenvatinib. The inclusion criteria were as follows: (1) patients who were receiving lenvatinib treatment for the first time and who had not received systemic therapy prior to this study; (2) intermediate-stage patients; (3) patients with a tumor burden that was beyond the up-to-seven criteria; and (4) CP A liver function. Patients were excluded if they (1) had received a systemic therapy other than lenvatinib (in other words, lenvatinib treatment was the second-line (n=22) or third-line treatment (n=4)); (2) were in an advanced stage (n=27) or an early stage (n=2) of the disease; (3) had CP B liver function (n=8); (4) were within the up-to-seven criteria (n=6); (5) received a therapy other than RFA or lenvatinib (i.e., resection, n=1; hepatic arterial infusion chemotherapy, n=2) after our initial lenvatinib treatment; and (6) the initial lenvatinib treatment was not started with the recommended dose (n=7), the patient received an inadequate course (using less than two weeks) (n=2), or if the treatment was presumed to have failed due to progressive disease (PD) obtained as the initial tumor response (n=3) (**Figure 1**). After the selection by these inclusion and exclusion criteria, 25 patients remained. Of them, nine patients received (initial) lenvatinib and (sequential) RFA treatment (combined group). There were three requirements for participation: (1) the patient had to agree to undergo RFA treatment; (2) the patient had to have no RFA contraindications such as severe coagulopathy or a tendency towards bleeding; and (3) it had to be determined that RFA would be safe and supposedly effective in the selected patients, for example, the lesion(s) had to have a regular shape and margin, and the lesions could neither be located adjacent to the diaphragm, the main branch of large blood vessels, or the bile ducts, nor be too superficial or deep. The other 16 patients received lenvatinib alone during the overall course of treatment (monotherapy group). In this group, we excluded three more patients who had lesions with an irregular shape and/or margin, which was an exclusion criterion for the combination group when considering insufficient ablation. The lesions with irregular shapes or margins were believed to have a higher incidence of microvessel invasion and even recurrence (22). Based on these criteria, 13 patients remained in the monotherapy group. Of these 13 patients, 10 had lesions that were not suitable for RFA treatment, while the other 3 did not consent to RFA treatment.

The study protocol conformed to the ethical guidelines of the World Medical Association’s Declaration of Helsinki and was approved by our institutional review board (approval number B210600051; approval date: June 9th 2021).

### 2.2 Lenvatinib Administration and Follow-Up

Lenvatinib was administered to the patients orally. Briefly, the lenvatinib dose was determined according to body weight as follows: patients weighing <60 kg received 8 mg lenvatinib once daily, whereas those weighing ≥60 kg initially received 12 mg lenvatinib once daily.

We followed up with all of the enrolled patients every week during the first month after the prescription of the initial dose of lenvatinib. Thereafter, patients with AEs that were Grade 2 or higher had to interrupt lenvatinib administration or decrease the dose to control the AEs; they were followed up with every other week (twice per month) during the subsequent follow-up periods. Conversely, patients with no or low-grade AEs were examined once a month.

During lenvatinib administration, according to the guidelines for lenvatinib administration, the drug dose was reduced, or the treatment was interrupted in patients who developed Grade ≥3 AEs or any intolerable Grade 2 drug-related AEs. This was maintained until the symptoms resolved, as indicated on the package insert.

### 2.3.Treatment Procedure for Lenvatinib and RFA

RFA was performed percutaneously under ultrasonographic guidance. A 480-kHz generator (VIVA RF generator; STARmed, Gyeonggi, Korea; Arfa RF ablation system; Japan Lifeline, Tokyo, Japan) and a 17-gauge internally cooled, adjustable RF electrode (VIVA; STARmed, Gyeonggi, Korea; Arfa active electrode; Japan Lifeline, Tokyo, Japan) were used. The lengths of the active tips of the electrodes applied in this study were 1.0 cm, 1.5 cm, 2.0 cm, or 3.0 cm. The selection of the electrode and the tip length was based on the tumor size, tumor location, and the operator’s preference. Every procedure aimed to obtain an ablative margin of no less than 5 mm around the treated lesions. A post-operative contrast-enhanced ultrasound examination was undertaken to evaluate the adequacy of the ablation. Complete ablation was defined as no perfusion of the contrast agent into the ablative area (which completely covered the lesion area as a whole), showing a completely black appearance with a distinct boundary.

For patients in the combination group, additional RFA was performed approximately 3 months after the start of lenvatinib administration in order to avoid possible cumulative AEs. When CR could not be achieved after one or several sessions of RFA treatment and/or in cases where there were still too many lesions or lesions that were too large after “initial lenvatinib plus RFA” treatment, lenvatinib treatment was restarted at a relatively smaller dose (dose was decreased to 4 mg capsule and/or the medication interval was reduced from once daily to once every other day) in order to prevent local recurrence or distant metastases. Lenvatinib was discontinued 4 days prior to RFA treatment preparation, and if re-started, the start date was set at 7–10 days after RFA (wait for the liver enzymes returned to almost normal levels).

### 2.4 Imaging Examination and Evaluation of the Outcomes

All patients underwent an imaging examination, such as contrast-enhanced CT and/or MRI, within 8 weeks from the start of the lenvatinib treatment. In general, all of the patients underwent an imaging examination every 2 months. After a 1-year follow-up period, the follow-up imaging examinations could be performed at less frequent intervals (set at about every 3 months). These intervals were changed slightly to control any AEs. The targets of the observation were (1) tumor response, including CR, partial response (PR), PD, stable disease (SD), and objective response rate (ORR); (2) survival, including PFS and OS; and (3) AEs.

**Figure 2:**
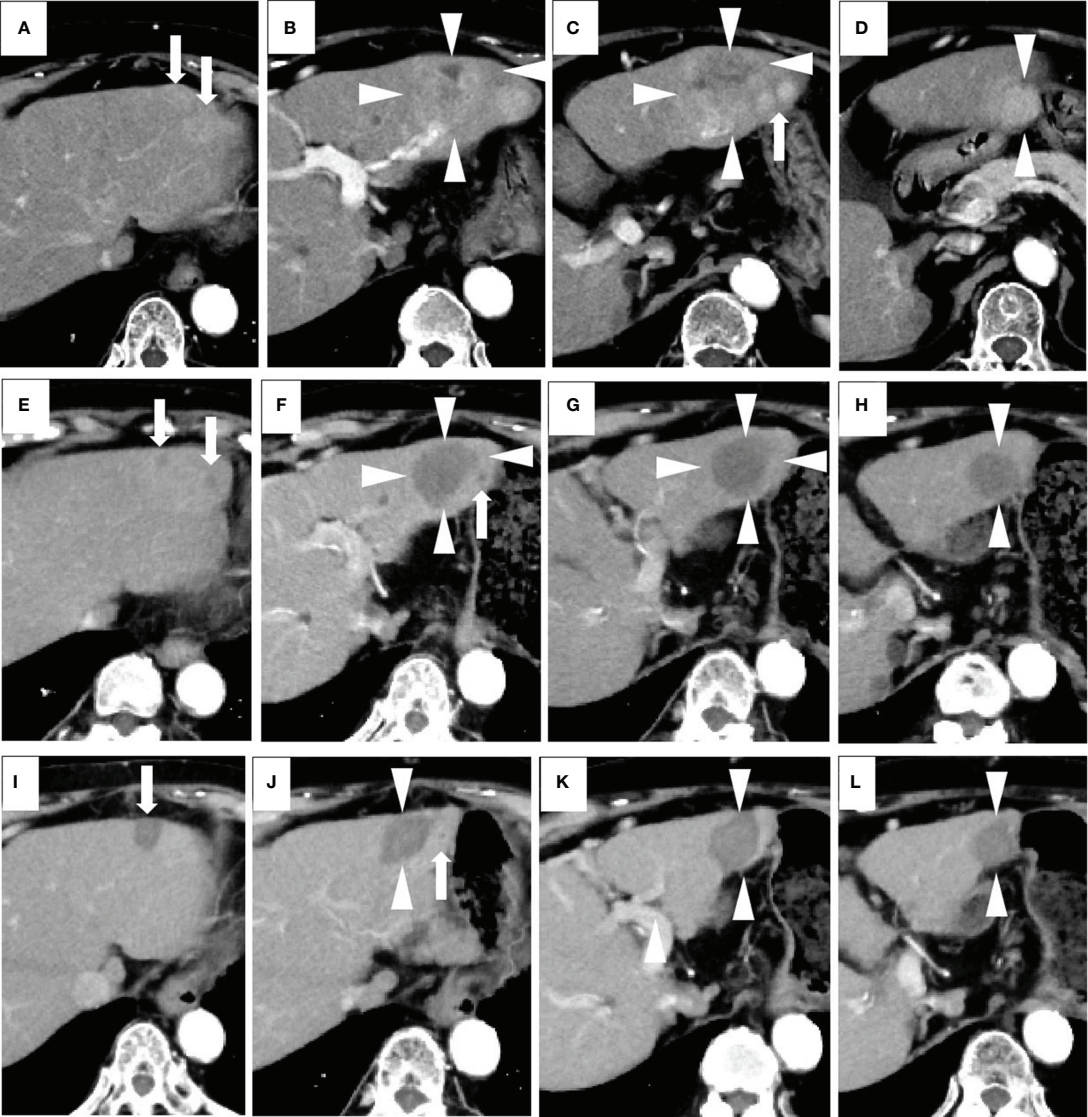
A case of combined lenvatinib and radiofrequency ablation (RFA) therapy. The patient was an 80-year-old female with no history of viral hepatitis. Her up-to-seven score was 9.3, and her Child-Pugh score was A5. All of these images were taken *via* arterial phase of contrast-enhanced CT (CECT). Images **(A–D)** were taken prior to the start of lenvatinib therapy. The target lesions were a larger one (a maximum diameter of 53 mm) (arrowheads) and a surrounding smaller one (arrows) located in the left lobe of the liver. Both of the tumors appeared as high-density areas. Images in **(E–H)** were taken 2 months after the administration of the recommended dose of lenvatinib. A marked decrease in the vascularity and size of each lesion were revealed. No newly developed lesions were observed. Therefore, this patient was evaluated as having a partial response according to the modified Response Evaluation Criteria in Solid Tumors (mRECIST). The maximum diameter of the main tumor decreased from 53 to 38 mm (arrowheads). One month after RFA, none of the lesions had any vascularity, and no new lesions were detected. Therefore, this patient was evaluated as having a complete response according to the mRECIST (image not shown). Images in **(I–L)** were taken 7 months after RFA (corresponding to 10 months from the start of lenvatinib), and all HCC lesions appeared as non-enhanced areas and showed a continual and marked decrease in size. Therefore, a complete response was recorded. For this patient, the initial tumor response was partial response (PR), while the best tumor response was complete response (CR).

**Figure 3:**
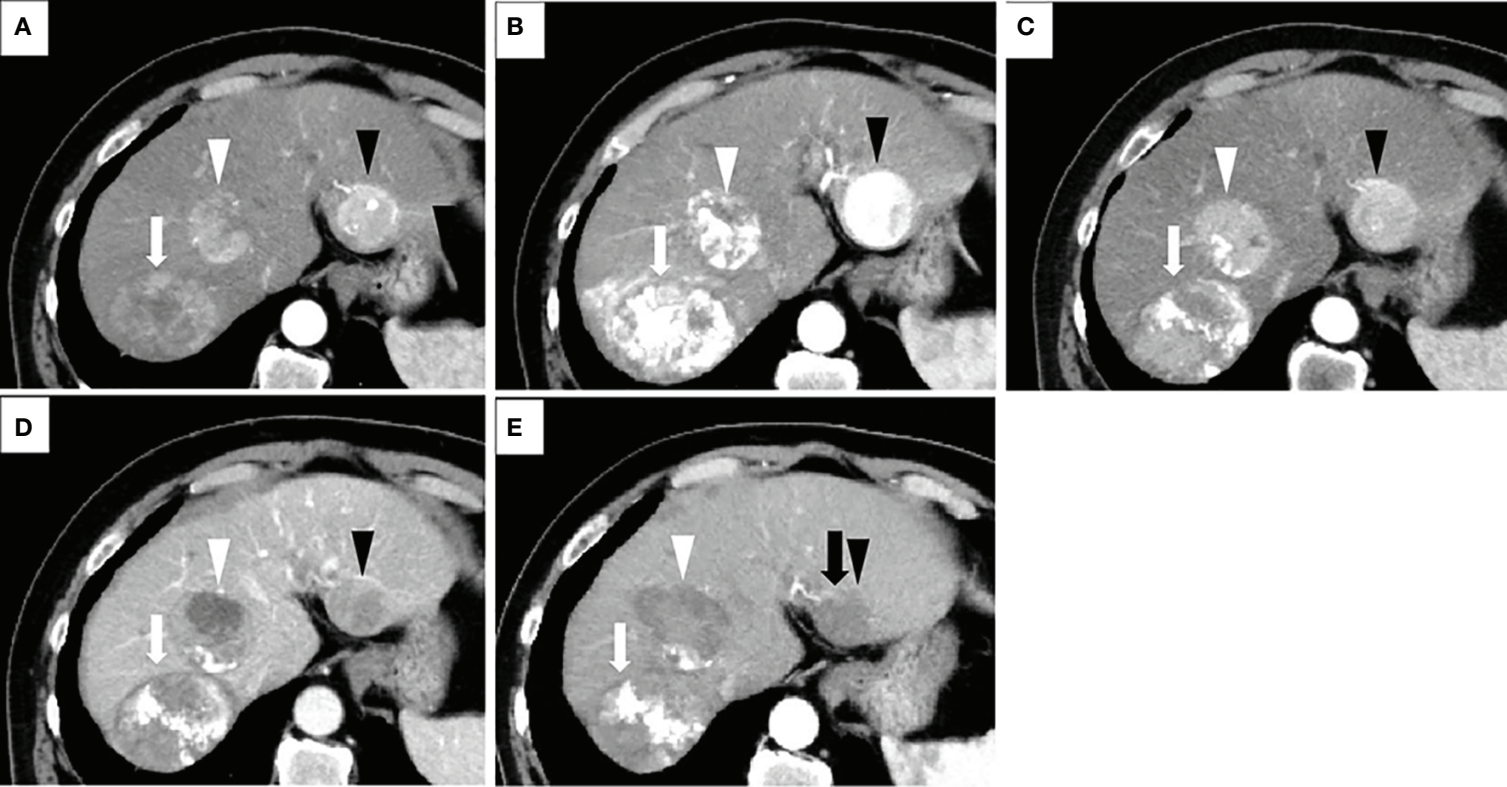
Treatment process of a patient in the combination group who had a transcatheter arterial chemoembolization (TACE) refractory history. The patient was a 68-year-old male with no history of viral hepatitis. At the time of his initial HCC diagnosis, the sizes of the lesions were 80mm (located on segment 7 of the liver and shown as white arrows), 50mm (S4, white arrowheads), and 40mm (S2, black arrowheads). His up-to-seven score was 11, and his Child-Pugh score was A5. All of these images were taken *via* arterial phase of contrast-enhanced CT (CECT). Image in **(A)** was taken prior to the start of TACE therapy. Afterwards, this patient underwent TACE treatment three times. The images in **(B, C)** were taken one and four months after the third TACE operation, respectively. The sizes of the three lesions in image **(C)** do not show many changes when compared to their size at initial detection **(A)**, suggesting TACE refractory. We changed the treatment plan from TACE to lenvatinib. One and half months after lenvatinib administration, a marked decrease in tumor vascularity was observed in image **(D)**, while the maximum diameter of the target lesion did not change compared to its pretreatment tumor size. No newly developed lesions were observed. Therefore, this patient was evaluated as having a partial response (PR) according to the modified Response Evaluation Criteria in Solid Tumors (mRECIST). After that, three radiofrequency ablation (RFA) operations were performed. Image **(E)** shows that two weeks after the third RFA, all of the lesions had decreased in size, and no new lesions were detected. However, the peritumoral hyperenhancement (black arrow) of the lesion in S2 (black arrowhead) indicates a residual viable tumor. Therefore, this patient was evaluated as experiencing PR according to the mRECIST. For this patient, both the initial tumor response and the best tumor response for our combination therapy were PR.

Based on the modified Response Evaluation Criteria in Solid Tumors (mRECIST) (23), we evaluated all of the contrast-enhanced CT and/or MRI images for tumor response. The tumor response was evaluated several times throughout the treatment procedures and thereafter during follow-up (**Figures 2** and **3**). The initial response was defined as the response determined on the basis of the imaging examination obtained within 8 weeks from the start of the initial administration of lenvatinib. In this setting, the best overall response was defined as the best response based on the imaging examination during this study. ORR was defined as the proportion of patients who experienced a PR or CR to the therapy, which is considered to be a direct measurement of a drug’s tumoricidal activity (24). Tumor response was evaluated *via* the consensus of two radiologists (with 7 and 20 years of experience in radiology, K.N. and S.K.). The radiologists were blinded to the patients’ demographic information.

PFS was defined as the time from lenvatinib initiation to the first occurrence of disease progression or death from any cause, whichever occurred first. OS was defined as the interval from lenvatinib to the last visit or the day of death, regardless of the cause of death. Patients who were lost to follow-up were censored at the last date they were known to be alive, and patients who remained alive were censored at the time of data cutoff.

Regarding the RFA procedure, treatment-related AEs were evaluated by serum alanine transaminase (ALT), aspartate transaminase (AST), and clinically visible complications (constitutional symptoms). For the combined lenvatinib and RFA treatment, clinically visible complications were recorded and graded according to the Clavien–Dindo classification (25). Other AEs, such as hypertension, fatigue, and loss of appetite were recorded and graded according to the National Cancer Institute Common Terminology Criteria for Adverse Events version 4.03.

### 2.5 Statistical Analysis

Continuous variables, when normally distributed, are described as the means and standard deviation, while when non-normally distributed data are presented as medians and range. Categorical variables are expressed as whole numbers. Two-tailed unpaired *t*-tests were performed to compare the continuous variables that were normally distributed; otherwise, the Mann–Whitney U-test was used. The differences in the distribution of the categorical variables between the two groups were analyzed using Pearson’s Chi square test or Fisher’s exact test. Survival analyses, including PFS and OS, were conducted *via* the Kaplan–Meier method with log-rank tests. Univariate and multivariate analyses were conducted using the Cox proportional hazards model to identify any risk factors that were associated with PFS and OS. In detail, dichotomous variables were dummy coded for entry into the regression model. Variables were included in the final model if the p-value at each iteration was below 0.1. All statistical analyses were performed with SPSS software (version 24.0, IBM Corp., Armonk, NY, USA). All p-values were based on two-sided statistical analyses, and p<0.05 was considered significant. The statistical analyses were performed by a statistician who was blinded to the patients’ actual treatment categories.

## 3 Results

### 3.1 Patient Characteristics

The baseline values of the combination group and the monotherapy group are summarized in **Table 1**. The majority of enrolled patients were male (18/22, 81.8%) and aged 76.1 ± 6.7 years old. The mean diameter of the largest lesion for all patients was (47.7 ± 31.8) mm. A total of 63.6% (14/22) patients had undergone treatment(s) other than systemic treatment before this study. The interval between previous treatment(s) and our initial lenvatinib treatment was at least three months. The patient and lesion baseline characteristics were similar between the two groups (all p>0.05). The treatment procedures of individual patients in the combination group are listed in **Supplementary Table 1**. Patient number 5 had a single lesion. All of the lesions were treated with RFA, except for those in patient number 2, for whom only some of the lesions (6 out of 11) were treated with RFA.

**Table 1:** Patient and lesion characteristics at the time of study entry.

Baseline characteristics	All patients (n = 22)	Monotherapy (n = 13)	Combination (n = 9)	p-value
*Patient variables*				
Sex (male/female)	18/4	10/3	8/1	0.474
Age (years, mean ± S.D.)	76.1 ± 6.7	76.1 ± 7.5	76.1 ± 5.7	0.991
Etiology (HCV or HBV/NBNC)^1^	10/12	7/6	3/6	0.342
Child–Pugh score (A5/A6)	16/6	9/4	7/2	0.658
mALBI grade (1/2a/2b)	11/5/6	5/4/4	6/1/2	0.388
AFP (ng/mL, median, range)^2^	16.5 (2–12400)	68.1 (2.7–12400)	12 (2–555)	0.171
Previous treatment for HCC^3^ (yes/no)	14/8	9/4	5/4	0.512
*Lesion variables*				
Tumor size^4^ (mm, mean ± S.D.)	47.7 ± 31.8	41.1 ± 22.9	57.2 ± 41.2	0.308
Lesion number (median, range)	4(1–12)	5(2–7)	4(1–12)	0.564
Tumor distribution (left lobe/right lobe/L and R)	2/4/16	1/2/10	1/2/6	0.868

^1^Only two patients in the monotherapy group were diagnosed with hepatitis B, while none of the patients in the combination group were. Because the number of patients with hepatitis B was so low, we combined the etiology of HBV and HCV for statistic.

^2^The AFP variable in both groups is not normally distributed.

^3^Previous treatments included resection, RFA, radiotherapy, TACE and hepatic arterial infusion chemotherapy, and any treatment other than systemic therapy.

^4^For multiple lesions, the tumor size indicates the diameter of the largest lesion, while for a single lesion, the tumor size is the diameter of this single lesion.

HBV, hepatitis B virus; HCV, hepatitis C virus; NBNC, non-HBV non-HCV; mALBI, modified albumin–bilirubin; AFP, alpha-fetoprotein; S.D., standard deviation.

### 3.2 Tumor Response and Survival

The follow-up time ended on February 22, 2022. The median follow-up period was 17.2 (6.7–38.5) months for all patients. The best overall responses for all patients in the combination group were obtained after RFA treatment. **Table 2** shows the best overall response and the initial response according to mRECIST. In the combination group, the number (rate) of patients with CR and PR as the best response was five (55.6%) and four (44.4%), respectively. The ORR in the combination group (100%) was slightly higher than that in the monotherapy group (76.9%). After the initial lenvatinib treatment, no patient displayed a CR. However, the PR rate of the combination group (66.7%) was higher than that of the monotherapy group (53.8%), but there no significant differences were observed (p = 0.548).

**Table 2:** Responses to the treatment according to the mRECIST.

Response Category	Combination (n = 9)	Monotherapy (n = 13)	p-value
*Best overall response*			
CR/PR/SD	5(55.6%)/4(44.4%)/0(0%)	0 (0%)/10 (50%)/3 (39.3%)	/
ORR	9 (100%)	10 (76.9%)	/
*Initial response*			
CR/PR/SD	0 (0%)/6 (66.7%)/3 (33.3%)	0 (0%)/7(53.8%)/6 (46.2%)	0.548

mRECIST, the modified Response Evaluation Criteria in Solid Tumors; ORR, objective response rate; DCR, disease control rate; CR, complete response; PR, partial response; SD, stable disease; PD, progressive disease. Data are presented as n and percentages (%).

**Figure 4:**
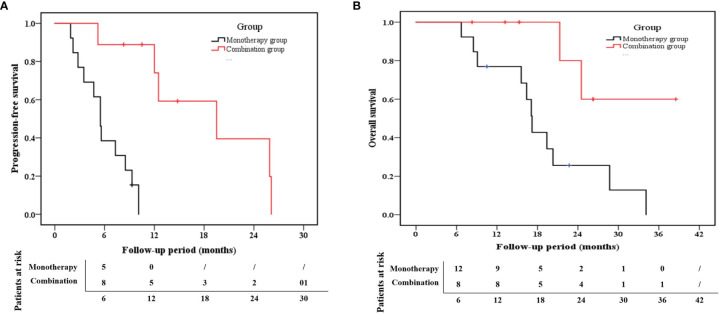
Kaplan–Meier estimates of **(A) **progression-free survival (PFS) and **(B)** overall survival (OS) for the combination group and the monotherapy group.

The median PFS for the combination group and monotherapy group was 12.5 months (95% confidence interval (CI): 9.3–20.7) and 5.5 months (95% CI: 4.2–7.6), respectively (**Figure 4**). Likewise, the 1-year PFS rate in the combination group (74.1%) was much higher than that in the monotherapy group (0%) (p < 0.001). With respect to the OS rate, the median OS was 21.3 months (95% CI:14.0–28.0) for the combination group and 17.1 months (95% CI: 12.6–22.2) for the monotherapy group (**Figure 4**). Additionally, the 1-year OS rates in the combination and monotherapy groups were 100% and 66%, respectively. The 2-year OS rate in the combination group (80%) was significantly higher than that in the monotherapy group (25.6%) (p =0.022).

As seen in **Table 3**, the univariate analysis demonstrated that the high AFP (>200 ng/mL) at baseline and the monotherapy treatment strategy were independent risk factors for PFS. Accordingly, multivariate Cox regression indicated that lenvatinib monotherapy (HR:18.22, 95% CI: 1.98–167.41, p =0.010) was the independent risk factor for PFS. For the OS analysis, the AFP and treatment strategy were selected once again *via* univariate analysis as variables for multivariate analysis. None of the indicators that we selected for this study was independent risk factors for OS (p>0.05, **Table 4**).

**Table 3:** Univariate and multivariate analyses of factors associated with PFS.

Variable	Univariate Analysis	Multivariate Analysis		
	p-value	HR	95% CI	p-value
Etiology (“HBV or HCV”/NBNC)^1^	0.503			
Age^2^ (≤70/>70 years)	0.407			
Sex (female/male)	0.872			
Child–Pugh class^2^ (A5/A6)	0.233			
AFP^2^ (≤/>200ng/mL)	0.037	0.42	0.13–1.40	0.158
Previous treatment for HCC^3^ (yes/no)	0.070	0.38	0.11–1.38	0.142
Tumor distribution (left lobe/right lobe/L and R)	0.662			
mALBI grade^2^ (1/2a/2b)	0.127			
Tumor size^2^ (≤3/>3 cm)	0.391			
Lesion number^2,4^ (1/2–6/>6)	0.493			
Treatment strategy (monotherapy/combination)	<0.001	18.22	1.98–167.41	0.010

^1^The result of this row compares NBNC with other etiologies (HBV and HCV).

^2^ The values of these variables were all recorded at the time of pre-treatment.

^3^Previous treatments included resection, RFA, SBRT, TACE, and hepatic arterial infusion chemotherapy as well as any treatment other than systemic therapy.

^4^For multiple lesions, the tumor size indicates the diameter of the largest lesion, while for a single lesion, the tumor size is the diameter of this single lesion.

PFS, progression-free survival; HBV, hepatitis B virus; HCV, hepatitis C virus; NBNC, non-HBV non-HCV; mALBI, modified albumin–bilirubin; AFP, alpha-fetoprotein; HR, hazard ratio.

**Table 4:** Univariate and multivariate analyses of factors associated with OS.

Variable	Univariate Analysis	Multivariate Analysis		
	p-value	HR	95% CI	p-value
Age^1^ (≤70/>70 years)	0.354			
Sex (female/male)	0.061	2.53	0.59–10.78	0.209
Etiology (HBV or HCV/NBNC)	0.690			
Child–Pugh class^1^ (A5/A6)	0.307			
Previous treatment for HCC^3^ (yes/no)	0.118			
Tumor distribution (left lobe/right lobe/L and R)	0.447			
AFP^1^ (≤/>200 ng/mL)	0.041	0.39	0.12–1.29	0.123
mALBI grade (1/2a/2b)	0.207			
Tumor size^1,2^ (≤3/>3cm)	0.750			
Lesion number^1^ (1/2–6/>6)	0.272			
Treatment strategy (monotherapy/combination)	0.022	3.79	0.80–17.98	0.094

^1^The values of these variables were all recorded at pre-treatment.

^2^For multiple lesions, the tumor size indicates the diameter of the largest lesion, while for a single lesion, the tumor size is the diameter of this single lesion.

^3^Previous treatments included resection, RFA, SBRT, TACE, and hepatic arterial infusion chemotherapy as well as any treatment other than systemic therapy.

OS, overall survival; HBV, hepatitis B virus; HCV, hepatitis C virus; NBNC, non-HBV non-HCV; mALBI, modified albumin–bilirubin; AFP, alpha-fetoprotein; HR, hazard ratio.

### 3.3 Safety

#### 3.3.1 AEs During All Treatment Course

For all of the patients enrolled in the study, no treatment-related deaths or Grade 4 AEs occurred. As seen in **Table 5**, the types of AEs that occurred in both groups were hypertension, appetite loss, fatigue, proteinuria, hypothyroidism, hand or foot skin reaction, AST/ALT elevation, and a worse modified albumin–bilirubin (mALBI) grade. There were no statistical intergroup differences for these AEs (all p>0.05) accept for ALT elevation (p=0.007). For diarrhea and rash, the two most common lenvatinib treatment related AEs, one case of diarrhea was occurred in the combination group, one case of rash was observed in the monotherapy group. Both AST and ALT elevation occurred in 89% of the patients in the combination group. The mALBI grade was detected to be slightly worse in less than half of the patients in the two groups, and no intergroup statistical differences were observed (p=0.937). The complications are listed and graded in **Table 5**.

**Table 5:** The types and grades of AEs in both groups.

	Combination group (n = 9)				Monotherapy group (n = 13)				p-value
	Grade 1	Grade 2	Grade 3	Total	Grade 1	Grade 2	Grade 3	Total	
Hypertension	0	4 (44.4%)	0	4 (44.4%)	0	8 (61.5%)	1 (7.7%)	9 (69.2%)	0.245
Appetite loss	3 (33.3%)	4 (44.4%)	0	7 (77.8%)	2 (15.4%)	4 (30.8%)	0	6 (46.2%)	0.138
Fatigue	4 (44.4%)	0	1 (11.1%)	5 (55.6%)	2 (15.4%)	3 (23.1%)	1 (7.7%)	6 (46.2%)	0.665
Proteinuria	0	1 (11.1%)	0	1 (11.1%)	0	2 (15.4%)	0	2 (15.4%)	0.774
Hypothyroidism	0	1 (11.1%)	0	1 (11.1%)	0	1 (7.7%)	0	1 (7.7%)	0.784
Diarrhea	1 (11.1%)	0	0	1 (11.1%)	0	0	0	0	/
Hand or foot skin reaction	2 (22.2%)	0	0	2 (22.2%)	1 (7.7%)	1 (7.7%)	0	2 (15.4%)	0.683
Rash	0				0	1 (7.7%)	0	1 (7.7%)	/
Temperature increase	4 (44.4%)	0	0	4 (44.4%)	0				/
AST elevation	1 (11.1%)	4 (44.4%)	3 (33.3%)	8 (88.9%)	7 (53.8%)	0	0	7 (53.8%)	0.083
ALT elevation	6 (66.7%)	1 (11.1%)	1 (11.1%)	8 (88.9%)	3 (23.1%)	1 (7.7%)	0	4 (30.8%)	0.007
mALBI grade elevation^1^	/			4 (44.4%)	/			6 (46.2%)	0.937

^1^Here, the elevation of the mALBI grade was the difference between mALBI at the end of the treatment as a whole and mALBI at the beginning of the initial lenvatinib treatment.

AEs, adverse events; AST, aspartate transaminase; ALT, alanine transaminase; mALBI, modified albumin–bilirubin. Data are presented as n and percentages (%).

#### 3.3.2 AEs Caused by the RFA Procedure Alone

All of the patients in the combination group were admitted to the hospital to be closely monitored for possible intraoperative and postoperative AEs due to RFA. Only one type of AE occurred after RFA, but not during lenvatinib treatment, that is fever. Four patients in the combination group experienced mild or moderate increases in body temperature after RFA treatment (4/9, 44.4%). As seen in **Supplementary Table 2**, the mALBI grade was assessed at different time-point treatment courses, the mALBI grade showed slight improvement one month after RFA (there was only one case where a worse mALBI grade was observed) when compared to the mALBI grade before RFA treatment(three cases showed a worse mALBI grade). However, no statistical differences were observed (p=0.257).

## 4 Discussion

Our research specifically targeted HCC lesions with relatively good liver function but with a high tumor burden (B2-HCC). The treatment of these lesions is indefinite and problematic because the tumors are neither metastasized nor localized enough to make an obvious choice regarding recommended treatments (26). However, it is worth exploring the best therapeutic strategy for B2-HCC because those in this population are believed to be potential candidates for multiple or combination therapies, with a possible curative intent (26). To the best of our knowledge, our study is the first to combine RFA and lenvatinib for HCC, especially for intermediate-stage HCC treatment. As specific measures do not currently exist to estimate what dose or time-point should be set, or what efficacy and safety outcomes would be obtained from this combination therapy, especially for intermediate-stage patients who have mild hepatic impairment, our attempt was challenging. However, because of this, the promising results of our research will undoubtedly be a valuable reference for further clinical research and applications.

In our study, both groups yielded a high best tumor response value (ORR = 76.9% and 100% for the monotherapy and combination groups, respectively), which is in good agreement with previous studies on lenvatinib administration with a similar enrolled population to our study (intermediate-stage patients for whom the reported ORR was 61.3% (27) or 73.3% (8) in different studies). These results consistently demonstrate the effectiveness of lenvatinib for the treatment of intermediate-stage patients. Lenvatinib may inhibit multiple tumoral angiogenesis process, such as *via* blocking both the VEGF receptor and the FGF receptor, decreasing the circulating endothelial cells, and decreasing the levels of angiopoietin 2 (which is specific for lenvatinib rather than sorafenib) (13). When the vascularity of the lesions decreases, in theory, cancer cells are less likely to invade and metastasize through the bloodstream. When combined with treatments other than RFA (TACE, hepatic arterial infusion chemotherapy, sorafenib, regorafenib, etc.), lenvatinib has been reported to be a more favorable treatment option over TACE as a first-line treatment for intermediate-stage HCC (8).

In our study, a higher tumor response rate was observed in the combination group (the CR rate as the best response was 55.6% and the PR rate as the initial response was 66.7%) than in monotherapy group. In addition, a promising survival effect was shown in the combination group in terms of the 2-year OS (25.6% vs. 80% for the monotherapy vs. combination groups; p<0.001). Kim et al. (28) reported that for patients undergoing multiple-session TACE treatment, patients with CR or PR as the initial response or the best response had a relatively longer OS. In agreement with this finding, for intermediate-stage HCC patients with preserved liver function (a similar patient population to ours), Park et al. (29) found that both the initial and best responses during repeated TACE were significantly associated with OS. According to these viewpoints, tumor response and OS are closely related, and our good results regarding the tumor response may positively influence a favorable prognosis. Furthermore, RFA first positively affected the tumor response rates in the short term and, by improving the tumor response, had a good influence on the long-term survival prognosis for B2-HCC CP A patients when administered as an add-on treatment.

RFA was introduced for intermediate-stage HCC patients in our study, which was partially due to the multi-lesion characteristics of our target patient population. For multi-lesion cases, local ablation on the liver was believed to have a positive effect on distant HCC lesions. Eros et al. found that conducting RFA on one HCC lesion would suppress the growth of a co-existing distant tumor, as the distant tumor’s size decreased significantly (30). Recently, the synergistic effect of RFA and the systemic therapy drug sunitinib (tyrosine kinase inhibitors, equivalent to lenvatinib) was observed for the treatment of both HCC mice and patients (31). This combined treatment was confirmed to significantly increase the frequency of CD8+T cell, memory CD8+ T cell, and dendritic cells; decreased the frequency of regulatory T cells; and activated tumor-specific antigen immune response in the tumor microenvironment. According to this theory, the addition RFA treatment, as was the case in our study, could represent a plausible therapy for CP A B2-HCC rather than repeated used of lenvatinib alone. Excitingly, our study obtained many positive results that were obtained due to the addition of RFA. Firstly, the best response for all patients in the combination group were obtained after RFA treatment. Secondly, the combination group exhibited significantly better PFS and OS rates than the lenvatinib monotherapy group, regardless of the median value or 1-year and 2-year rates (p<0.05). More importantly, monotherapy (when compared with the combination treatment) was determined to be a risk factor for PFS.

One of the highlights of our research strategy is that we used lenvatinib as a first-line systemic treatment rather than a sequential treatment after RFA. We planned this procedure based on the following considerations: First of all, lenvatinib can inhibit HCC cell progression and proliferation by activating the FGF signaling pathways, which may reduce tumor size. Because of the reduction in lesion size, the tumor may be downstaged, facilitating the use of more curative treatments for early-stage HCC (27). RFA is widely acknowledged as the first-line curative option for unresectable BCLC early-stage HCC. Hence, the treatment strategy of lenvatinib followed by RFA may adhere to the international consensus to the fullest extent possible. Secondly, for intermediate-stage HCC, which characterized by large and/or multiple lesions, multi-needle and/or multi-site ablation would be required. Nevertheless, the multi-needle and/or multi-site ablation would increase the risk of surgical bleeding and surgical complications. Reducing the tumor’s blood supply and/or decreasing the size *via* lenvatinib treatment is undoubtedly advantageous for reducing the risks of the RFA operation and to achieve a sufficient ablative zone. It is worth mentioning that our original intention was to achieve the most curative treatment for HCC possible through our designed combination therapy. In terms of the encouraging tumor response results (100% ORR including 55.6% CR), which indicated cancer shrinkage or disappearance after treatment, we have achieved our curative intent.

In order to minimize AEs, we carefully set an appropriate time interval for the combination therapy in our experimental design. Pharmacokinetic analysis revealed that, generally, the terminal elimination half-life of plasma lenvatinib is approximately 28 hours (32). Lenvatinib is mostly metabolized in the liver. Unlike other types of solid tumors or in a healthy population, patients with HCC have a more or less reduced liver function that possibly decreases lenvatinib clearance, and HCC patients may not well tolerate lenvatinib at a higher plasma concentration (33). Out of a consideration for AEs that potentially accumulated during the combination treatment, lenvatinib administration was interrupted 4 days before RFA, the time-point of which was estimated based on the total clearance of plasma lenvatinib. Generally, serum AST and ALT levels, a reflection of the underlying liver metabolic burden, would be elevated when the hepatocytes are injured and become necrotic through various causes, such as hepatotoxic drugs (lenvatinib oral administration), and physical injury, including hyperthermia caused by RFA (34). Our study revealed that the majority of patients (88.9%, 8/9) in the combination group had elevated serum ALT and AST. What is worse, the incidence of a patient having an advanced ALT grade was much higher in the combination group (p=0.007). To avoid the cumulative liver injury AEs caused by lenvatinib and RFA, we specifically restarted lenvatinib treatment about 7–10 days after RFA (when the ALT and AST were supposed to decrease to almost normal levels). Fortunately, no serious AEs (over grade 3) presented themselves during the course of our study, which may indicate the safety of the combination therapy presented here. There were no statistical inter-group differences in the mALBI, nor any statistical differences before and after RFA treatment. As ALBI is a well-recognized and objective biomarker for hepatic reserve function (35), this result may suggest that additional RFA performance would not increase the risk of aggravated hepatic function.

The limitations of our study should be acknowledged. First, the patients were not randomly assigned to the combination and monotherapy groups during grouping. Only patients who were suitable and on whom it would be safe to perform RFA operation were included in the combination group. Seen from the opposite direction, however, this design was essential to ensure patient safety during the ablation procedure. To solve the problem of possible heterogeneity due to the non-randomized grouping, we recorded inter-group statistics regarding the baseline characteristics of the patients and lesions, and no differences were found. Second, the number of patients analyzed was small since it was difficult to enroll suitable CP A B2-HCC patients to receive our novel treatment. The small sample size might be the reason why we had a slightly shorter PFS (the median value of the monotherapy group was 5.5 months) compared to the published data from the REFLECT trial (the median value of PFS for lenvatinib was 7.4 months) (9). To overcome this problem, a large-scale study, preferably a multicenter study, would be valuable to prove our results.

## 5 Conclusion

In conclusion, our innovative combination treatment plan using first-line systemic lenvatinib therapy and subsequent RFA might be a potential option for patients in the intermediate stages of HCC with good liver function but a heavy tumor burden, as demonstrated by the better tumor response, improved rates of survival, and non-inferior safety when compared to the use of lenvatinib alone. Further clinical trials with longer follow-up periods and large-scale population are expected to confirm these findings in the near future.

## Data Availability Statement

The raw data supporting the conclusions of this article will be made available by the authors, without undue reservation.

## Ethics Statement

The studies involving human participants were reviewed and approved by the Ethics Committee of Yokohama City University Medical Center (protocol code B210600051; date of approval: 9 June 2021). The ethics committee waived the requirement of written informed consent for participation.

## Author Contributions

Conceptualization, KN; methodology, SK; software, SMo; validation, HM; formal analysis, LR; investigation, KO; resources, AN; data curation, SMa; writing—original draft preparation, FW; writing—review and editing, FW and KN; visualization, MC; supervision, KS; project administration, SK; funding acquisition, n/a. All authors have read and agreed to the published version of the manuscript.

## Funding

This research was supported by the National Natural Science Foundation of China (No. 82102074) Key Science and Technology Program of Shaanxi Province of China (No. 2022SF-320).

## Conflict of Interest

The authors declare that the research was conducted in the absence of any commercial or financial relationships that could be construed as a potential conflict of interest.

## Publisher’s Note

All claims expressed in this article are solely those of the authors and do not necessarily represent those of their affiliated organizations, or those of the publisher, the editors and the reviewers. Any product that may be evaluated in this article, or claim that may be made by its manufacturer, is not guaranteed or endorsed by the publisher.

## Supplementary Material

Supplementary Table 1 The treatment procedures in the combination group. ^1^Treatment process means the course of treatment, which is from the begging of initial lenvatinib administration to the allover follow-up endpoint of this study, which was February 22, 2022. ^2^For patients No. 1, 5, 7, 8, and 9, lenvatinib administrated was continued until the end of the follow-up period. For patient No. 2, lenvatinib was re-administered was used until lenvatinib was discontinued due to adverse events. ^3^For multiple lesions, the tumor size indicates the diameter of the largest lesion, while for a single lesion, the tumor size is the diameter of this single lesion. RFA, radiofrequency ablation; HCC, hepatocellular carcinoma; CECT, contrast-enhanced CT; CR, complete response; AEs, adverse events.

Supplementary Table 2 Evaluation of mALBI in combination group^1^. ^1^mALBI, modified albumin–bilirubin; N, no change; RFA, radiofrequency ablation.
