# Supplementary material for: Combination Therapy With Lenvatinib and Radiofrequency Ablation for Patients With Intermediate-Stage Hepatocellular Carcinoma Beyond Up-To-Seven Criteria and Child–Pugh Class A Liver function: A Pilot Study
Source: Front Oncol. 2022 May 4;12:843680. doi: 10.3389/fonc.2022.843680 (PMC9114706; doi:10.3389/fonc.2022.843680)
Supplement: Supplementary Table 1 — The treatment procedures in the combination group. 1Treatment process means the course of treatment, which is from the begging of initial lenvatinib administration to the allover follow-up endpoint of this study, which was February 22, 2022. 2For patients No. 1, 5, 7, 8, and 9, lenvatinib administrated was continued until the end of the follow-up period. For patient No. 2, lenvatinib was re-administered was used until lenvatinib was discontinued due to adverse events. 3For multiple lesions, the tumor size indicates the diameter of the largest lesion, while for a single lesion, the tumor size is the diameter of this single lesion. RFA, radiofrequency ablation; HCC, hepatocellular carcinoma; CECT, contrast-enhanced CT; CR, complete response; AEs, adverse events. [file Table_1.docx]

**Supplementary Table 1. The treatment procedures in the combination group.**

| **Patient No.** | **Treatment process ^1,2^** | **Tumor size^3^ (mm)** | **Lesions treated by RFA (n and percentages)** | **Main reason for stopping lenvatinib or introducing RFA treatment** |
| --- | --- | --- | --- | --- |
| 1 | Lenvatinib+ 2 times RFA+Lenvatinib | 95 | 2 (100%) | Residual viable HCCs were continually identified by CECT after a long period of lenvatinib administration. Other therapy was needed. |
| 2 | Lenvatinib+RFA+Lenvatinib | 19 | 6 (54.5%) | With repeat treatment by lenvatinib, the patient presented with several kinds of AEs, which resulted in dose reduction. However, size of some lesions increased hereafter. Other therapy was needed. |
| 3 | Lenvatinib+RFA+Lenvatinib | 135 | 2 (100%) | Appetite loss relating lenvatinib was observed, which resulting in dose reduction. Other therapy need to be introduced. After RFA and then additional lenvatinib, cerebral infarction (not related to lenvatinb and RFA) was observed. The treatment for HCCs including lenvatinib administration was stopped. |
| 4 | Lenvatinib+2 times RFA | 25 | 6 (100%) | Patient refused to take lenvatinib treatment after a period of lenvatinib administration. Other therapy need to be introduced. After treated by RFA twice, CR were obtained for 6 lesions. Therefore, no additional lenvatinib was done after RFA treatment. |
| 5 | Lenvatinib+ 2 times RFA+Lenvatinib | 70 | 1 (100%) | After lenvatinib treatment, tumor size and/ or vascularity were decreased after lenvatinib treatment, which enables RFA to operate safely and efficiently, that is, to have practicability (Before lenvatinib treatment, the lesion was bigger than 3 cm or more than 4 lesions). RFA was applied in the consideration of curable intend.  For No. 6 patient, CR were obtained for all the lesions after RFA treatment. In addition, several AEs related to lenvatinib occurred during initial lenvatinib administration. Therefore, no additional lenvatinib was done after RFA treatment. |
| 6 | Lenvatinib+RFA | 53 | 4 (100%) |  |
| 7 | Lenvatinib+RFA+Lenvatinib | 19 | 7 (100%) |  |
| 8 | Lenvatinib+4 times RFA+Lenvatinib | 19 | 12 (100%) |  |
| 9 | Lenvatinib+ 3 times RFA | 80 | 3 (100%) |  |
