# Supplementary material for: Combination Therapy With Lenvatinib and Radiofrequency Ablation for Patients With Intermediate-Stage Hepatocellular Carcinoma Beyond Up-To-Seven Criteria and Child–Pugh Class A Liver function: A Pilot Study
Source: Front Oncol. 2022 May 4;12:843680. doi: 10.3389/fonc.2022.843680 (PMC9114706; doi:10.3389/fonc.2022.843680)
Supplement: Supplementary Table 2 — Evaluation of mALBI in combination group1. 1mALBI, modified albumin–bilirubin; N, no change; RFA, radiofrequency ablation. [file Table_2.docx]

**Supplementary Table 2. Evaluation of mALBI in combination group^1^.**

| **Patient No.** | **mALBI grade**  **(1/2a/2b)** | | |  | **mALBI grade change**  **(worsened/( no change or improved))** | | **p value** |
| --- | --- | --- | --- | --- | --- | --- | --- |
|  | **At start of lenvatinib administration** | **Before RFA** | **One month after RFA** |  | **Before RFA** | **One month after RFA** |  |
| Total | 6/1/2 | 3/0/6 | 2/3/4 |  | 3/6 | 1/8 | 0.257 |
